# Temporal Super-Resolution Using a Multi-Channel Illumination Source

**DOI:** 10.3390/s24030857

**Published:** 2024-01-28

**Authors:** Khen Cohen, David Mendlovic, Dan Raviv

**Affiliations:** The Faculty of Engineering, Department of Physical Electronics, Tel Aviv University, Tel Aviv 69978, Israel; mend@eng.tau.ac.il

**Keywords:** computational photography, super-resolution, temporal super-resolution, active illumination

## Abstract

While sensing in high temporal resolution is necessary for a wide range of applications, it is still limited nowadays due to the camera sampling rate. In this work, we try to increase the temporal resolution beyond the Nyquist frequency, which is limited by the sensor’s sampling rate. This work establishes a novel approach to temporal super-resolution that uses the object-reflecting properties from an active illumination source to go beyond this limit. Following theoretical derivation and the development of signal-processing-based algorithms, we demonstrate how to increase the detected temporal spectral range by a factor of six and possibly even more. Our method is supported by simulations and experiments, and we demonstrate (via application) how we use our method to dramatically improve the accuracy of object motion estimation. We share our simulation code on GitHub.

## 1. Introduction

Resolution in a digital signal refers to its frequency content. High-resolution (HR) signals are band-limited to a more extensive frequency range than low-resolution (LR) signals. While sampling a signal, the captured signal’s resolution is limited by two factors: the physical device limitation (e.g., the device’s response function to different frequencies) and the sampling rate. For example, digital image resolution is limited by the imaging device’s optics (the diffraction limit) and the sensor’s pixel density (the sampling rate).

Super-resolution is a broad research area that uses sophisticated ways to overcome these limits. The ability to exceed the resolution limits of the system always has something to do with some prior knowledge about the scene or about the system [1,2]. In the field of imaging, image super-resolution (SR) techniques can be divided into two main approaches: optical-based and algorithm-based.

Optical-based SR utilizes the optical property of light to transcend over the diffraction limit. This approach can be further divided into mainly three areas: the first is multi-plexing spatial-frequency bands [1], which uses the fact that low-frequency moire fringes are formed when the scene is multi-plexed with a periodic pattern (structured illumination)—e.g., the work by Abraham et al. in speckle structured illumination [3]. The second involves acquiring multiple parameters about the scene and merging them, for example, detecting scene polarization [4]. The third method is the probing near-field electromagnetic disturbance method. It is a modern approach that uses an unconventional imaginary optical system and tries to detect tiny disturbances in electromagnetic waves. For example, using evanescent waves [5]. Each of these super-resolution methods sacrifices another domain [1,2,6]. For example, on account of the time [7], wavelength [8,9], or field of view [10].

Algorithm-based SR is a method that focuses on the sensor pixels’ density limit. It includes mainly algorithmic solutions, such as frame deblurring and localization estimators [11]. Nowadays, deep learning methods have presented excellent performance in SR tasks [12,13,14], including medical imaging [15], satellite imaging [16], and face SR [17].

The field of temporal super-resolution (TSR) deals with a similar challenge but in the temporal domain. In general, TSR can be divided in a similar manner: optical-based and algorithm-based. Optical-based TSR includes several methods. One is s combination of cameras: this method exploits the fact that different cameras with some temporal overlap can provide complementary information to increase the temporal resolution. The temporal coding method uses a preknown temporal pattern as a coding technique for the detected signal. Optical coding extracts temporal illumination patterns [18] or temporarily coded apertures [19], and sensor coding uses a temporary change in the sensor’s reading manner [20] or a flattened shutter [21] to deblur the images. Software Interpolation uses algorithms (nowadays, these are mainly deep learning-based) to generate a temporal interpolation of the signal. Some of the methods are optical flow-based [22], whereas others are phase-based [23] or kernels-based methods [24,25].

Algorithm-based TSR (Software-only) approaches a straightforward solution in terms of system complexity, and these methods demonstrate good performance [26]. However, their ability to interpolate in time is limited since the deep learning models heavily rely on past examples and training. In contrast, TSR supported by hardware (optics or sensor) has the potential to raise the temporal sampling frequency with a much higher rate and reliability. However, the price is the complexity of the system.

While spatial super-resolution has been widely researched for decades, temporal super-resolution (TSR) has not been extensively researched to the same extent. As a consequence, there is still plenty of room for improvement in TSR methods, especially ones that provide a high up-sampling factor, high reliability, and low system complexity.

In this work, we present a novel approach to TSR by using the object’s optical reflection properties, such as its surface polarity reflection or spectral reflection. Our proposed system consists of a standard camera with a high-frequency illumination source. In comparison to other presented methods, our approach constitutes a good compromise between performance (large temporal spectrum reconstruction) and simplicity (a system that is not too complicated or expensive). We model the camera image sensor operation method, formulate our problem as an optimization problem, and provide a comprehensive solution for a particular case of colored-based illumination sources. Our analysis and results are supported by theoretical derivations, simulations, and experimental results. Apart from other works in this field, our method shows high reliability in terms of spectral reconstruction with no significant hardware complexity penalty, and it reconstructs the spectral content of the signal very well. Moreover, our method can be used in real time due to its simple solution form.

The main contributions of this work are as follows:

1. The demonstration of a novel approach for optical coding to achieve high temporal frequencies with a fixed sensor sampling rate working in real time.
2. The development of a substantial theoretical background to increase temporal resolution from subsamples.
3. Providing an anti-aliasing algorithm to improve system performance over a wide range of frequencies.

## 2. Theoretical Background

### 2.1. Temporal Model for an Image Sensor

We denote a general signal as I(x,y,t), captured by the image sensor. We formulate the image sensor operation as temporal distortion, which is assumed to be linear time-invariant (LTI), followed by sampling. The distortion is represented by the transfer function, h(t), and the sampling in time at a frequency of 1 over the exposure time is $f_{s}=\frac{1}{T}$.

The sampled signal, therefore, is given by

(1) $$u\left[n\right]\equiv u\left(t=nT\right)=\left[\sum_{n=-\infty }^{\infty }\delta \left(t-Tn\right)\right]\left[f(t)*h(t)\right]=\sum_{n=-\infty }^{\infty }\delta \left(t-Tn\right){\left[f(t)*h(t)\right]}_{t=nT}$$

In order to fully reconstruct the u(t) signal, two conditions have to be fulfilled: first, the distortion of the signal can not be too severe, and the sampling rate must be at least twice as high as the maximum spectral content of u(t), according to the Shannon-Nyquist theorem of sampling [27]. It is clear that effectively increasing the sampling rate can improve the signal reconstruction in the temporal domain. However, since it means that the sampling rate becomes much higher, the integration time decreases, which, in turn, leads to the signal-to-noise (SNR) degradation of the reconstruction signal.

### 2.2. Multi-Channel Approach and Assumptions

We define a set of channels as a set of independent optical properties of light. For example, X-polarization and Y-polarization are two channels, or several different wavelengths are different channels. We assume linear optics, meaning that the reflected light from an object does not transform between channels. We define the channel *m* as follows:

(2) $$C^{m}=\int_{0}^{T}\int_{-\infty }^{\infty }c^{m}\left(\lambda ,t\right)Q^{m}\left(\lambda \right)R\left(\lambda ,t\right)d\lambda dt$$

While c(λ,t) is the illumination mask generated by a light source (changes in time), R(λ,t) is the reflective properties of the object, (change in time) and Q(λ) is the image sensor filter for a specific spectral range; λ is the wavelength, and *T* is the integration time of the sensor (exposure time).

We assume that for a given *m*, there is a spectral match between the flicker light source and the sensor filter. Practically, it means that the sensor is significantly affected by the light source (e.g., a red bulb will be captured intensively in the camera’s red channel). In addition, we assume that $c^{m}\left(\lambda ,t\right)$ is a product of a temporal-dependent function and a spectral-dependent function:

(3) $$c^{m}\left(\lambda ,t\right)Q^{m}\left(\lambda \right)=c^{m}\left(t\right){\tilde{Q}}^{m}\left(\lambda \right)$$

Moreover, we assume that there is a high similarity between the different channels. So, for each time *t*, the relation between the light collected in each channel is equal up to a constant scale, γ:

(4) $$\int_{-\infty }^{\infty }{\tilde{Q}}^{m}\left(\lambda \right)R\left(\lambda ,t\right)d\lambda \approx \gamma^{m,k}\int_{-\infty }^{\infty }{\tilde{Q}}^{k}\left(\lambda \right)R\left(\lambda ,t\right)d\lambda$$

Now, we focus on the case where the flicker changes in time in a discrete manner between two modes: off and on. Therefore, we get

(5) $$C^{m}=\sum_{n=1}^{N}c_{n}^{m}\int_{-\infty }^{\infty }{\tilde{Q}}^{m}\left(\lambda \right)R\left(\lambda ,t\right)d\lambda \approx \sum_{n=1}^{N}c_{n}^{m}i_{n}$$

where *N* is the up-sampling factor and $i_{n}$ represents the average value of the image at a subtime step, *n*.

### 2.3. Definitions

In our analysis, we denote *N* as the up-sample factor of the sampling rate, meaning that we increase the maximum detected spectrum from $\frac{1}{2T}$ to $\frac{N}{2T}$, and *M* is the number of independent channels that we used. We assume that in any sub-interval of time, $\frac{T}{N}$ is approximately constant, so for each exposure time, we can define the intensity vector of size *N*, $\vec{I}$. We further define the vector $\vec{C}$ of size *M* to represent the value captured in each of the channels for a single exposure time. We define M vectors (*m* is between 1 to *M*) for the vectors ${\vec{c}}^{m}$ of size *N* to represent each of the channel’s code patterns. In our analysis, we focus on the cases where the vectors ${\vec{c}}^{m}$ have binary values, 0 or 1, when the flicker of the channel, *m*, is on or off, respectively.

## 3. Method

From Equation (5), one can notice that extracting the values of $i_{n}$ is equivalent to an up-sample in factor N in the temporal domain. The problem is that for *M* channels, this equation can be solved uniquely only for an up-sample factor of N=M. In practice, we have a low number of channels, and we want to get a high rate of temporal super-resolution. For that, we need to use some prior knowledge about the scene dynamics. We choose to assume scene smoothness in the temporal, so we formulate the problem as the following cost function:

(6) $$L=\sum_{n=1}^{N-1}{\left(i_{n}-i_{n+1}\right)}^{2}+\sum_{m=1}^{M}\lambda^{m}\left(C^{m}-\sum_{n=1}^{N-1}i_{n}c_{n}^{m}\right)$$

where $\lambda^{m}$ represents some regularization factors.

### 3.1. Spatial Regularization

**Figure A1:**
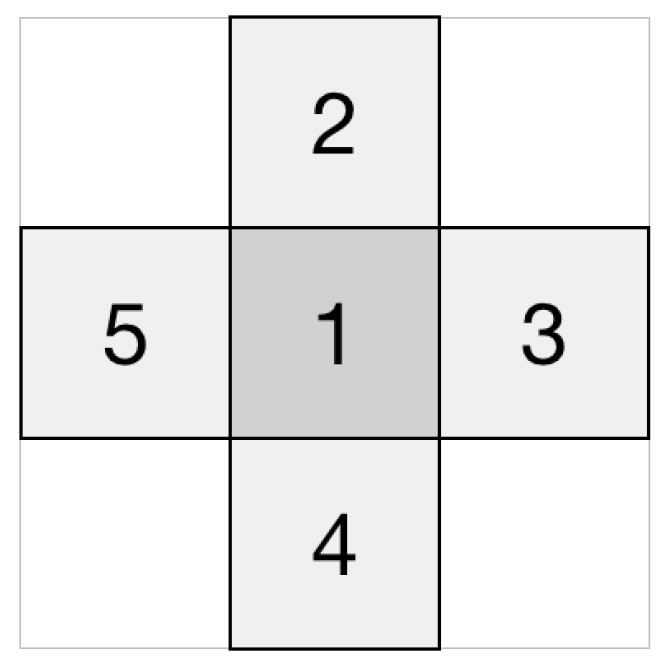
Pixel neighbor order, as was used in our method. The P domain includes pixels 2, 3, 4, and 5.

The absence of any spatial correlation between adjacent pixels might yield some artifacts in the image. To avoid this, we define (for each pixel) a domain *P*, which includes the pixel with its four closest neighbors (see Figure A1 in Appendix A.3), and we modify the cost function as follows:

$$\begin{array}{c} L=\sum_{n=1}^{N-1}\sum_{x,y\in P}w_{x,y}^{t}{\left(i_{x,y,n}-i_{x,y,n+1}\right)}^{2}+w_{x,y}^{s}{\left(i_{x,y,n}-i_{x,y+1,n}\right)}^{2}+\\ w_{x,y}^{s}{\left(i_{x,y,n}-i_{x+1,y,n}\right)}^{2}+\sum_{m=1}^{M}\sum_{x,y\in P}\lambda_{m,x,y}\left(C_{m,x,y}-\sum_{n=1}^{N}i_{x,y,n}c_{x,y,m,n}\right) \end{array}$$

The vectors change to become column stack vectors of the different pixels, and the matrices are expanded to block matrices, as explained in Appendix A.3. *w* factors are weight factors that determine the ratio between spatial and temporal regularization.

### 3.2. Solution with Lagrange Multipliers

Finding the solution of Equation (6) means that from the infinite number of solutions to Equation (5), we would like to choose the one with the smoothest solution to be the estimator for the actual signal. The solution is given by the following equation (for the complete derivation, please see Appendices Appendix A.1 and Appendix A.2):

(7) $$\vec{I}=M^{-1}S{\left(S^{⊺}M^{-1}S\right)}^{-1}\cdot \vec{C}$$

### 3.3. Colored Light Source

We focus on a particular case of flicker for the colors red, blue, and green. This case is the most common and can be used on any colored camera. We denote the flicker vectors as ${\vec{c}}^{1}=\vec{r}$, ${\vec{c}}^{2}=\vec{g}$, and ${\vec{c}}^{3}=\vec{b}$, and the channel vector is ${\vec{C}}^{⊺}=\left(R,G,B\right)$, while *R*, *G*, and *B* are the digital color values, as captured in the image for each color.

The spectral matching assumption (as presented) is fulfilled because the light source spectrum (LED spectrum) is captured well by the sensor’s color channels. This leads to the following interpretation (in digital values):

(8) $$u\left(t\right)\approx \gamma^{r}u_{r}\left(t\right)\approx \gamma^{g}u_{g}\left(t\right)\approx \gamma^{b}u_{b}\left(t\right)$$

While u(t) is the true signal and $u_{r}\left(t\right)$, $u_{g}\left(t\right)$, and $u_{b}\left(t\right)$ are the signals as captured in red, green, and blue, respectively. The γ factors are related to the color of the object and can be derived from a single image of the scene (without flicker).

**Figure 1:**
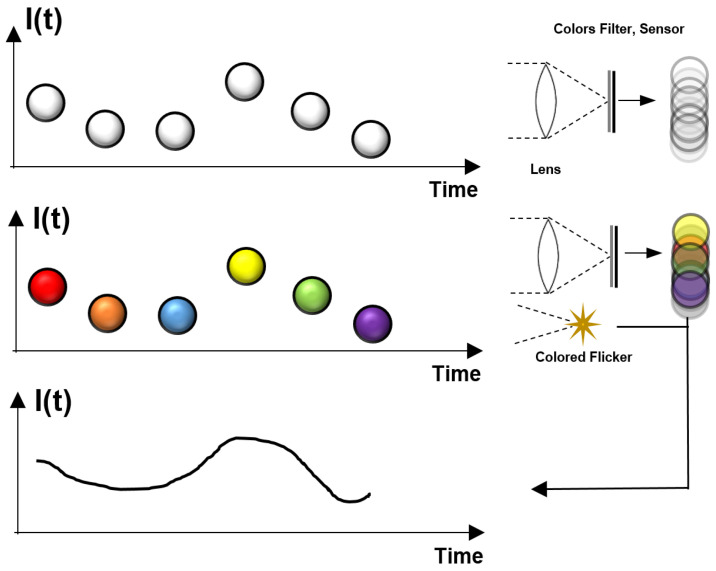
Schematic diagram of our method. An object moves and changes its intensity value under different illumination conditions: Top—arbitrary environment illumination. In this case, there is no obvious way to reconstruct the object intensity value in time since the sensor integrates all the light from the scene. Middle—under colored flicker illumination with our prior knowledge about the flicker pattern, we can recover the object intensity value with high-quality certainty. Among all the possible temporal profiles, we choose the most reasonable one in the sense of minimum energy. Bottom—the high-resolution reconstructed signal.

A binary flicker pattern of red, green, and blue for each camera’s exposure time illuminates the scene. The total accumulated result is used to extract the value of the actual signal (see Figure 1).

### 3.4. The Scanning Mode and Anti-Aliasing Algorithm

Since *N* represents the up-sample factor, the smaller the *N*, the more accurate the result should be (for N=3, the result is even unique), but no information about higher frequencies is collected. On the contrary, the high *N* factor can detect high-frequency content but is less reliable. Therefore, we propose a technique that applies several *N* factors, each at a separate temporal window. At the same time, we define a temporal window as a period in which the method works at a constant factor, *N*.

The construction of the signal is carried out in the spectral domain. However, collecting all the contributions from the different temporal windows is not straightforward. There could be many approaches to combination strategy. We chose the following: each spectral interval of the united signal is given by averaging over all the temporal windows’ contributions, with a minimum *N* factor that detects this spectral interval. For example, given a camera with an FPS of $f_{s}$, if we apply a scanning method with the sequence N=3,4,5,6, the low spectral domain (up to $3\frac{f_{s}}{2}$) is equal to the spectral content of the first temporal window, the mid-spectral domain (from $3\frac{f_{s}}{2}$ to $2f_{s}$) is equal to the spectral content of the second temporal window, and the high spectral content (from $2f_{s}$ to $5\frac{f_{s}}{2}$) is equal to the average between the third and fourth temporal windows.

One assumption that underlies this method’s basis is that the spectral content of the scene does not change much between temporal windows (invariant signal for a short time). According to that, choosing the shortest possible temporal windows is preferred, yet if the temporal windows are too short, this may not provide enough accurate results for the spectrum.

Because every temporal window contributes to another spectral component, an assembly between the windows can be used. However, anti-aliasing techniques should be used to avoid artifacts. Therefore, we can use mutual information from the different spectral domains to attenuate and even eliminate aliasing Algorithm 1.    
**Algorithm 1:** Anti-aliasing algorithm
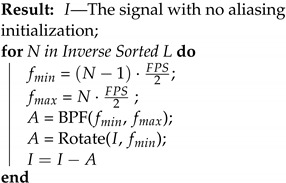


While BPF is an ideal band-pass filter, Rotate is a function that rotates the signal’s spectrum relative to a specific frequency. The algorithm uses the fact that every temporal window with a specific *N* is aliased mainly by the spectral components from the components of the N+1 temporal window. In this way, we use the spectrum that the temporal window had recovered with the up-sampling factor of N+1 and subtract its aliasing contribution from the spectral range recovered by the temporal window using the up-sampling factor *N*.

### 3.5. Performance Analysis and Signal-to-Noise Ratio (SNR)

As presented in previous sections, typically, increasing the FPS causes a decrease in exposure time. The SNR of the signal increases linearly with exposure time [28]. Hence, reducing the exposure time should decrease the SNR. However, the SNR grows like a square root in the illumination intensity (or the number of photons) [28]. Since our method uses an active illumination source, it compensates for the SNR decrease and improves image quality. We analyze the signal and the noise separately; see the Appendix for the extensive derivation Appendix A.6. The final result is as follows:

(9) $$SNR\propto \alpha^{3/2}$$

where we define α as the ratio between the intensity of the active illumination source and the intensity of the background source.

## 4. Numerical Simulations and Analysis

In order to demonstrate our method, we built a computational simulator. The simulator simulates an ideal matt and white object, with no environmental illumination, that performs any dynamics such that a particular pixel in the image can be described as a continuous trajectory of intensity versus time. Apart from the scene, the simulator simulates the camera sampling method via integration and sampling in FPS and effective flickers in RGB colors. Everything is assumed to be ideal such that in the presence of a red flicker (for example), there is no green and blue intensity value captured at all. Furthermore, we set the exposure time to be equal to one over the camera’s frames-per-second, neglecting the sensor reading time delay (which is a good approximation for common cases in reality). For each of the following results, unless otherwise mentioned, we simulated camera FPS at 10 Hz and we limited our analysis to the cases where N=3, N=4, N=5, and N=6, but this can be examined for higher up-sampling factors as well.

### 4.1. Flicker Pattern Analysis

The freedom to choose the flicker pattern raises the question of which pattern should be chosen to maximize the signal reconstruction performance. In other works, how to find the optimal coding [29] instance by assuming some noise model has been shown; however, here, we are interested in our method’s performance for different spectrum domains without any explicit assumptions made on the sample noise model. We later show how this spectral approach can be leveraged into a very high upsampling coefficient by using the anti-aliasing scheme (by decomposing the spectral components and using the optimal flickering pattern for each spectral domain).

For example, for a specific channel (B, G, or R), one can choose whether to perform a flicker at one specific time step and then get as much information as possible about this specific time step (at that channel) or apply the flicker to some time steps. Then, the camera collects the accumulative values of this channel, which has uncertainty about any specific time step. However, it gives information from a more extensive temporal range from the signal. Two approaches have been examined here: the reconstruction error for randomly changing patterns over time and a comparison between some arbitrary flicker patterns. For both analyses, we simulated 10,000 random sinus functions, with a temporal frequency of [5 Hz, 30 Hz] (uniformly distributed) and a total duration of 5 s each.

**Figure 2:**
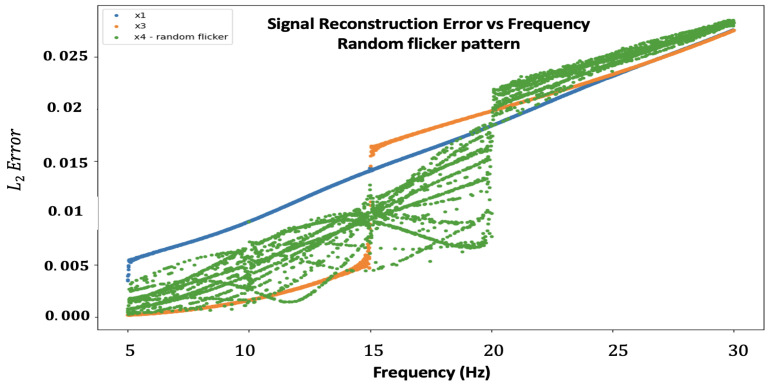
Signal reconstruction error vs. the frequency when using a random S full-rank matrix for each frame. The result (in green) is represented and compared to the non-up-sampled signal (blue) and the particular case of the reconstructed signal of N=3, and *S* is the identity matrix (orange).

Randomly Flicker: This analysis was carried out via the random sampling of the flickering pattern. Practically, we sample full-rank matrices, *S*, for each frame and calculate the method L2 error, and the results are shown in Figure 2. From our analysis, the random sampling has different errors for each spectral content. Therefore, we suggest using the random sampling technique when there is no prior knowledge about the scene frequency content.

Our second test was carried out to examine the reconstruction error for different fixed flicker patterns. Ideally, it is best to search among all the possible existing matrices, but this number is enormous, and we decided to focus on several specific flicker patterns for N=4, N=5, and N=6 (see Appendix A.5 to see the different choices).

**Figure 3:**
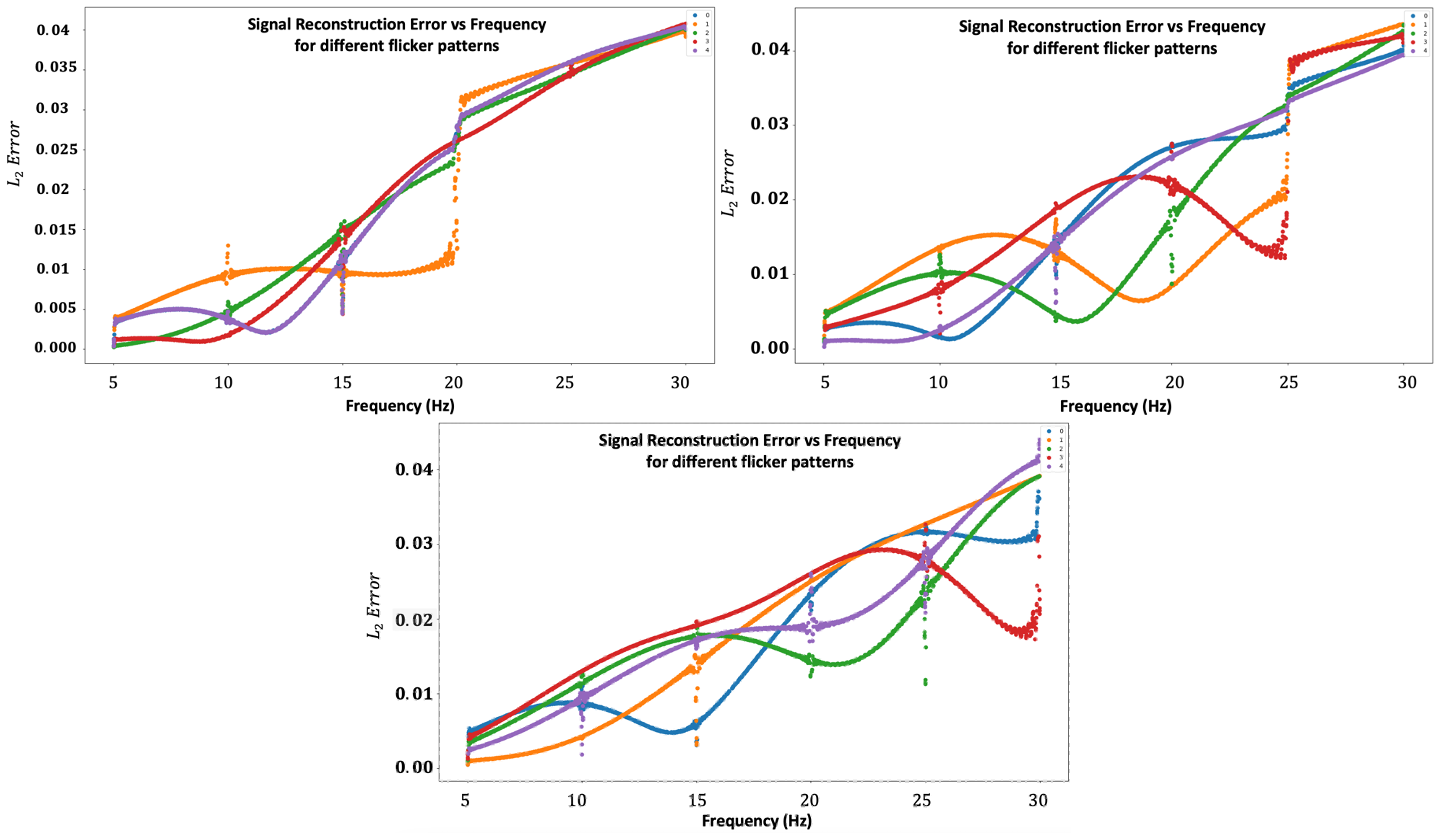
Signal reconstruction error comparison between several candidates for the flicker pattern for different up-sample factors, *N* (4—top left, 5—top right, and 6—bottom). The Y-axis represents the error, and the X-axis represents the frequency.

The results are represented in Figure 3. For each N factor, the “jump” in error at a certain frequency (20 Hz, 25 Hz, and 30 Hz, respectively) is due to the Nyquist theorem of sampling. For each *N*, there is no one specific graph that can be considered the best one among all candidates. Nevertheless, it is quite clear that if we focus on a specific spectral range, we can divide the spectrum into adjacent regions, where each N gets its lowest error. For example, N=3:[5 Hz, 15 Hz], N=4:[15 Hz, 20 Hz], N=5:[20 Hz, 25 Hz], and N=6:[25 Hz, 30 Hz], and due to this, we choose the best flicker pattern as follows: pattern 1 (for *N* = 4), pattern 3 (for *N* = 5), and pattern 4 (for *N* = 6). These results support our scanning method attitude for merging different temporal windows to construct the entire spectral domain.

An additional comparison is presented in Table 1.

### 4.2. Simulations Results

Here, we compare various *N* factors with the signal reconstruction L2 error. The generated signal was, as in the previous section, obtained via the random sampling of 10,000 sinus functions at temporal frequencies of [5 Hz, 30 Hz] (uniformly distributed). The flicker patterns we chose to use, based on previous analysis, are

$$\begin{array}{cc} N=3:\; & \vec{b}=\left(1,0,0\right),\;\vec{g}=\left(0,1,0\right),\;\vec{r}=\left(0,0,1\right)\\ N=4:\; & \vec{b}=\left(1,0,0,1\right),\;\vec{g}=\left(1,0,1,0\right),\;\vec{r}=\left(0,1,0,1\right)\\ N=5:\; & \vec{b}=\left(0,1,0,0,0\right),\;\vec{g}=\left(1,0,1,0,1\right),\;\vec{r}=\left(0,0,0,1,0\right)\\ N=6:\; & \vec{b}=\left(1,0,1,0,1,0\right),\;\vec{g}=\left(0,1,0,1,0,1\right),\;\vec{r}=\left(1,1,1,1,1,1\right) \end{array}$$

**Figure 4:**
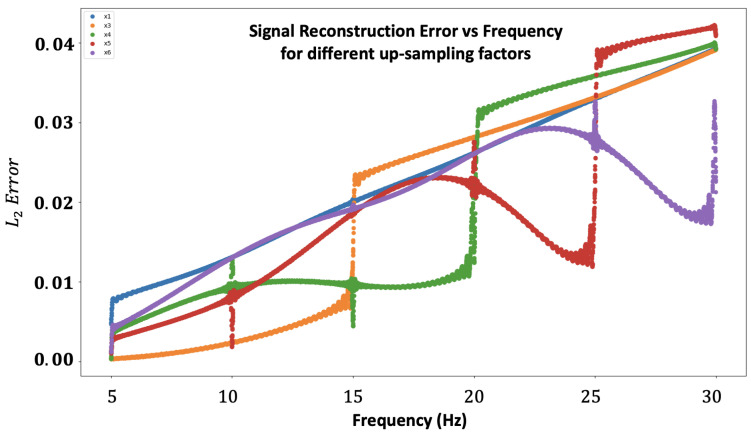
Signal reconstruction error between the actual harmonic signal vs. different frequencies. The Y-axis represents the error rate, and X-axis represents the frequency. A comparison between various up-sampled factors. A frequency of 5 Hz is the maximum the camera can detect due to the Nyquist theorem, an up-sample factor of *N* = 3, 4, 5, and 6 extends the frequency range to 15 Hz, 20 Hz, 25 Hz, and 30 Hz, respectively.

The results can be seen in Figure 4, where one can figure out several conclusions. First, the blue line (the linear curve) is the maximum error among all values, and it is given by the camera’s original signal with no up-sampling factor. Second, every up-sample factor extends the frequency detected range up to a different cut-off frequency because of the Nyquist sampling theorem. Third, the reconstruction quality for a different N is dependent on the frequency, whereas each up-sampling factor reaches better results in different frequency regions. This insight might help a lot when there is some prior knowledge about the scene spectrum. Moreover, these findings also support the scanning method technique we presented.

**Figure 5:**
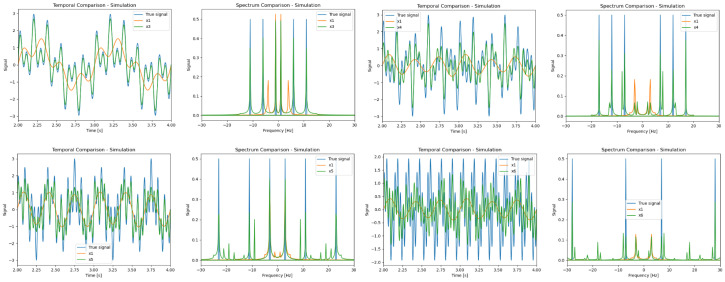
Simulation results for different signals; *N* = 3, 4, 5, and 6; camera FPS = 10. Blue is the original signal; Orange is the camera reconstruction (no TSR); Green is our TSR algorithm.

The simulation results are shown in Figure 5, where we simulated the following temporal signals:

$$\begin{array}{cc} N=3:\; & x(t)=\sin\left(2\pi t\right)+0.3\sin\left(4\pi t\right)+0.8\sin\left(10\pi t\right)+0.25\sin\left(18\pi t\right)+0.75\sin\left(22\pi t\right)+0.5\sin\left(36\pi t\right)\\ N=4:\; & x(t)=\sin\left(2\pi t\right)+\sin\left(12\pi t\right)+\sin\left(22\pi t\right)\\ N=5:\; & x(t)=\sin\left(6\pi t\right)+\sin\left(22\pi t\right)+\sin\left(46\pi t\right)\\ N=6:\; & x(t)=\sin\left(14\pi t\right)+\sin\left(56\pi t\right) \end{array}$$

and the following different flicker patterns:

$$\begin{array}{cc} N=3:\; & \vec{b}=\left(1,0,0\right),\;\vec{g}=\left(0,1,0\right),\;\vec{r}=\left(0,0,1\right)\\ N=4:\; & \vec{b}=\left(0,1,0,0\right),\;\vec{g}=\left(1,0,0,1\right),\;\vec{r}=\left(0,0,1,0\right)\\ N=5:\; & \vec{b}=\left(0,1,0,0,0\right),\;\vec{g}=\left(1,0,1,0,1\right),\;\vec{r}=\left(0,0,0,1,0\right)\\ N=6:\; & \vec{b}=\left(1,0,0,0,0,1\right),\;\vec{g}=\left(0,1,1,0,0,0\right),\;\vec{r}=\left(0,0,0,1,1,0\right) \end{array}$$

One can notice that our method significantly improves the ability to detect and reconstruct the signal spectral content even though one can still recognize the aliased signal parts.

In order to demonstrate this technique’s performance and the anti-aliasing algorithm results, we simulated a signal 10 s long, and we defined 2, 3, and 4 temporal windows, each at a size of 5 s, 3.33 s, and 2.5 s, respectively. Every temporal window had its own *N* factor.

The simulated signal is

(10) $$x\left(t\right)=SW_{12}\left(t\right)+SW_{19}\left(t\right)+SW_{23}\left(t\right)+SW_{27}\left(t\right)$$

**Figure 6:**
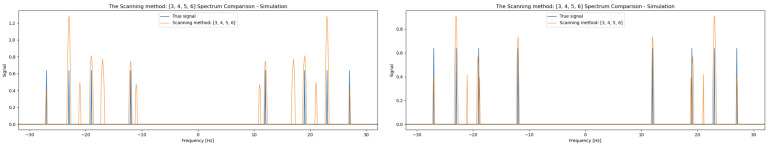
Our anti-aliasing algorithm in the scanning mode; combination of *N* = 3, *N* = 4, *N* = 5, and *N* = 6. Left: before the algorithm; right: after the algorithm. All aliasing was eliminated up to a frequency of 20 Hz.

While SW represents a square wave with a 50% duty cycle, we took the *N* factors to be 3, 4, 5, and 6 (each corresponds to a temporal window). The result is shown in Figure 6. To avoid white noise, we filtered out the lowest 5–10% of the spectrum (filter uniform in the spectrum).

We can see that we obtained a good result when using this technique and even an additional improvement when using the anti-aliasing algorithm.

## 5. Experimental Results

### 5.1. The Setup

**Figure 7:**
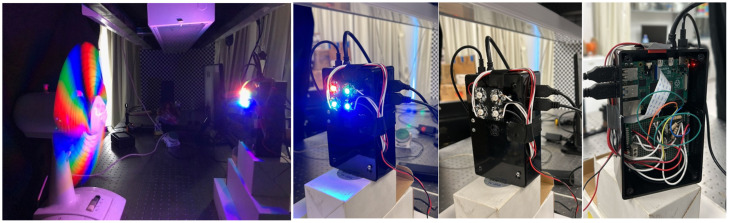
The experimental setup. Left: A rotating fan in front of our camera setup. Right: Our camera setup, with synchronized LEDs (one red, one blue, and two green) and a Raspberry Pi.

**Figure 8:**
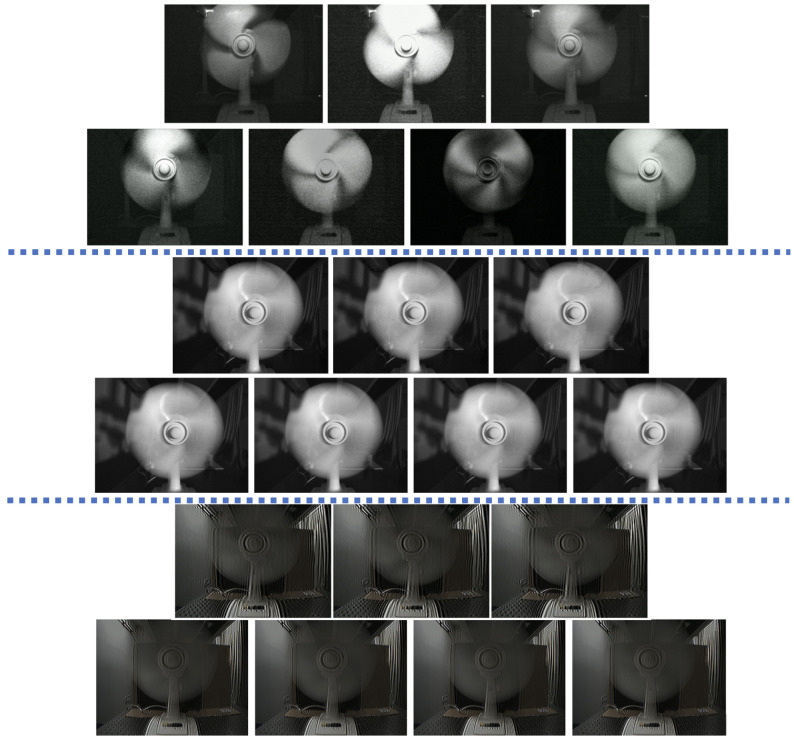
Different imaging examples for *N* = 3 and *N* = 4. Top: Our technique; Middle: SuperSlowmo [30]; Bottom: the Flatter Shutter technique [21]. Here, we used $W_{t}=3$ and $W_{s}=1$.

**Figure 9:**
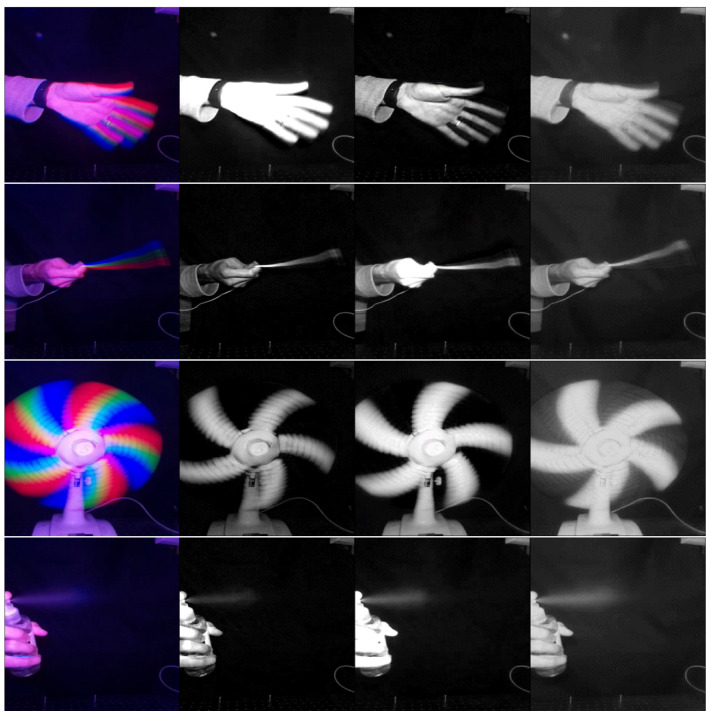
Basic examples of our up-sampling method for different scenes (each row). Here, we used N=3, while the first column (from left to right) shows the recorded frame, and the three other columns show the temporal sequence.

Our experimental setup can be seen in Figure 7. Our setup consists of a Raspberry PI unit with an RGB camera, a model Pi Camera V2 (which we set to a frame per second of 10 Hz, 20 Hz, or 80 Hz), four light bulbs (one red, two green, and one blue) and a power bank (22.5 Watt). We placed different objects in front of the camera at a typical distance of about 40 cm to 2 m. The main object we examined was the rotating fan since it allowed us to analyze different temporal frequencies (see Figure 8), but we also tried different objects (see Figure 9). The frequency of the rotating fan was measured in parallel by a recording camera (PointGrey) in high-FPS mode (with a typical frame rate of up to 500 Hz, limited to the region of interest); this measurement allowed us to compare our results to the ground truth. For the SNR measurement, we used white paper instead of the object. For every N (upsampling factor), we used the same coded pattern that was found to be the best among the candidates presented in the simulations (Figure 3). We set the rotational fan frequency to approximately ±21.5 Hz. In addition, we normalized the DC value for the different signals to focus only on temporal variations.

### 5.2. Signal Reconstruction Results

**Figure 10:**
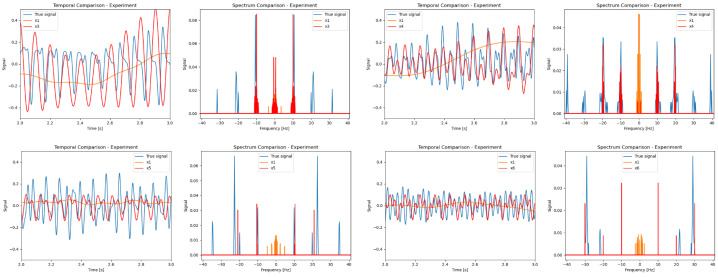
Experimental results for *N* = 3, 4, 5, and 6; camera FPS = 10. Blue is the original signal; orange is the camera reconstruction (no TSR); red is our TSR algorithm. Our method successfully detects spectral components up to a frequency of 30 Hz.

The experimental results are shown in Figure 10.

These results indicate that our method successfully detects high frequencies. Nevertheless, it can be seen from the graphs that sometimes there are some errors and artifacts in the result.

### 5.3. Imaging Reconstruction Results

**Figure A4:**
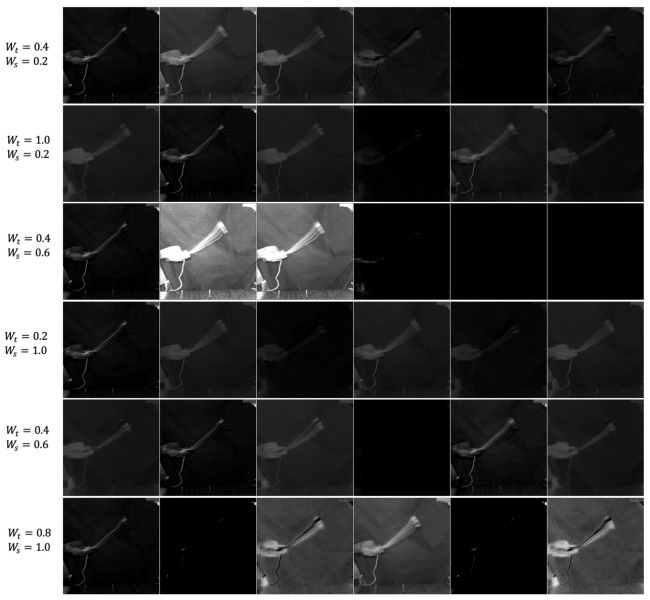
Rotating rope (counter clockwise) from left to right. Analysis for different temporal and spatial weights (*N* = 6).

Apart from the ability to capture high frequencies, Figure 8 and Figure 9 show the imaging results as examples. For comparison, we used the SuperSlowmo algorithm [30] to raise the frame-per-second rate of the scene. Moreover, we analyzed the imaging results for different temporal and spatial weights in Figure A4, Appendix A.7.

### 5.4. SNR and Performance Results

**Figure 11:**
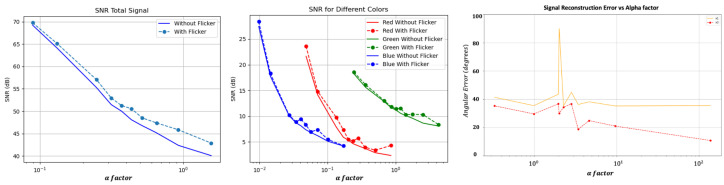
Left–Middle: SNR measurement with (left) and without (middle) flicker (vs. α factor). Please notice that α is on a logarithmic scale. Right: Experimental measurements of cosine similarity between the actual signal and the reconstructed one for different α values; note that α is in a logarithmic scale (N=3).

In order to evaluate the SNR for different α factors, we used a clean, white piece of paper located ±40 cm in front of the camera and the flicker. We used different environmental illumination by using a white-light projector and measured the illumination values using a Lux meter. The results are shown in Figure 11.

As one can notice, the SNR improved since the flicker increases the light in the scene.

An additional experiment was used to measure the performance of the method reconstruction vs. the α factor. Here, we focus on N=3, and the results are shown in Figure 11. In fact, there is a decrease in the performance of the method when the α factor decreases, which means increasing the illumination of the environment relative to the flicker illumination source.

### 5.5. Motion Estimation Improvement

**Figure 12:**
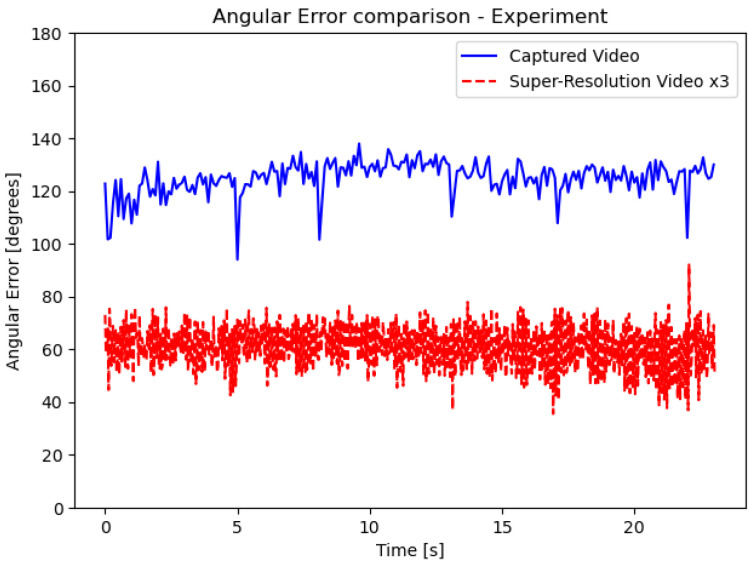
The rotating fan experiment; the original video vs. the up-sampled version video (N=3) error comparison regarding motion estimation with time [31]. The lighting condition is poor, and the detection task is difficult, but there is still a significant improvement in the ability to detect the proper motion.

One fundamental task in computer vision is to estimate motion or optical flow. Given the image’s spatial and temporal derivatives, one can calculate the velocity of a pixel in the XY plane. However, estimating the temporal derivative relies heavily on the camera frame-per-second rate. Here, we introduce an application for our method. Since high temporal frequencies cannot be detected in a low frame-per-second camera, applying our method and effectively raising the camera frame-per-second can improve the temporal aspect. We measured the rotating fan’s blade velocity (at the XY plane) at each pixel and compared it to the ground truth, which was detected using a high frame-per-second camera. The result is shown in Figure 12.

### 5.6. Motion Estimation Analysis

A comparison between motion estimation performance with and without our method is presented in Figure 12.

### 5.7. Discussion

The results demonstrate how our method can significantly increase the temporal upper limit of the camera. We have reached the following conclusions. First, the results demonstrate how our method can significantly raise the temporal upper limit of the camera. We found that applying different flickering patterns can deal with a significant change in the signal reconstruction error, and, as expected, the greater the *N* factor, the higher this error becomes. Additionally, it has been shown that each flicker pattern can provide better accuracy at a particular frequency when taking into account other frequencies. Following these findings, we introduced the scanning method, which has displayed good results, including aliasing attenuation. Experimentally and supported by theoretical derivation, our experiment shows how the SNR of the scene increases with our method. We demonstrated how our system performance improves when the α factor improves, as per Figure 11. The errors are still low, even at a low α (Figure 11). Furthermore, we show how the motion estimation error decreases dramatically when using our method (Figure 12). From the experimental and simulation results, it is clear that our method successfully detects high temporal frequencies.

However, our method still suffers from several issues and limitations, which influence the reconstruction error, and we divide them into three aspects:

(1) Illumination errors: Some of the assumptions about the light sources do not hold all the time, for example, the flickering illumination intensity differences that require tuning the coefficients $\gamma_{r}$, $\gamma_{g}$, and $\gamma_{b}$.

(2) Temporal mismatch: The better the synchronization between the camera and light source, the lower the signal reconstruction error will be, and the less artifacts will be seen.

(3) Reconstruction error: Our analysis has shown an inherent error factor in our method, especially for a high *N*. This error component might lead to the generation of new frequencies and signal distortions (as can be seen for N=6 in Figure 10).

## 6. Conclusions

In this work, we introduced a new method for temporal super-resolution based on multi-channel flickering light sources. We presented a method to solve the problem based on Lagrange multipliers. Our method showed very good results in our tests for several combinations of flickering patterns and up-sampling factors, *N*. We further demonstrated the performance of our scanning method and anti-aliasing technique. In our experiment, the results were good as well, and our method was able to extract very high frequencies (by a factor of about six) from the original camera Nyquist cut-off frequency. Moreover, we demonstrated (experimentally) how a motion estimation task is significantly improved thanks to our method. While achieving temporal super-resolution is always accompanied by a trade-off between the accuracy results and system complexity, here, we demonstrated a method that constitutes a proper balance between the two. As discussed in the previous section, despite the attractiveness of our method, it still has limitations, for example, the performance decrease for strong background illumination or the technical challenge of synchronizing the camera with the active light source. The up-sampling factor can go up to six (with moderate errors) and beyond without any significant overhead for the system hardware complexity. For future study, we suggest three directions: the first one is to examine different channel types, e.g., using different polarization. The second one is improving the reconstruction algorithm by taking into account different spatial and temporal correlations, and the third one is to examine the method performance for different noise models. To encourage future research, we share our code on GitHub.

## 7. Patents

A US patent has been submitted for this method.

## Figures and Tables

**Table 1:** Reconstruction error for each *N* factor for different frequency ranges.

*N*	Frequencies [Hz]	L2 Error [×$10^{-3}$]	Normalized Error
3	5–15	±3.1	1
4	15–20	±10	3.22
5	23–25	±14	4.5
6	28–30	±18	5.8

**Figure A2:**
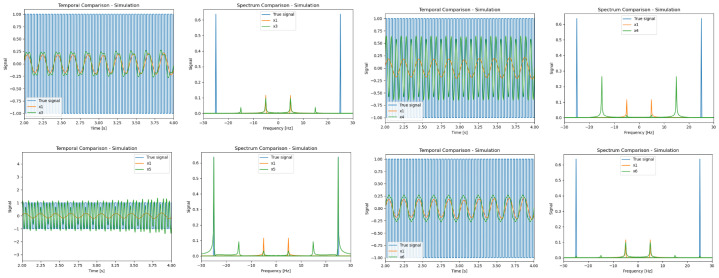
Simulation result comparison for a square wave with a 25 Hz base frequency. Comparison for different *N* factors: 3, 4, 5, and 6 in the order of top-left, top-right, bottom-left, bottom-right, respectively.

**Figure A3:**
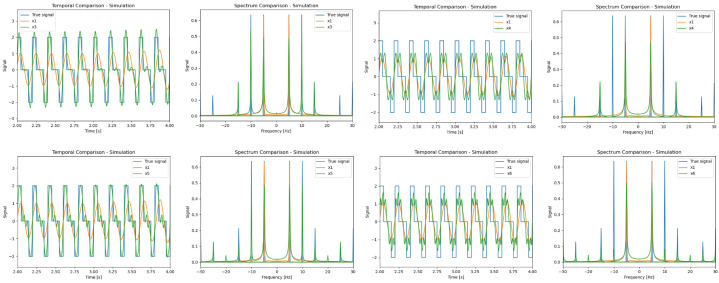
Simulation result comparison for a square wave with 5 Hz and 10 Hz base frequencies. Comparison for different *N* factors: 3, 4, 5, and 6 in the order of top-left, top-right, bottom-left, and bottom-right, respectively.

## Data Availability

The data presented in this study are available on GitHub or may be obtained from the authors upon reasonable request.

## Abbreviations

The following abbreviations are used in this manuscript:

TSR	Temporal super resolution
FPS	Frames per second
HR	High resolution
LR	Low resolution
SNR	Signal-to-noise ratio

## Appendix A

### Appendix A.1. Definitions

We define a vector in size M as representing the Lagrange multiplier of each of the channels $\vec{\lambda }$. Finally, we define the matrices S and M, which will be used in our derivations:

(A1) $$S_{\left(N,M\right)}=\left(\begin{array}{ccc} {\vec{c}}^{1} & \cdots & {\vec{c}}^{M} \end{array}\right),\;{\vec{C}}_{\left(M,1\right)}=\left(\begin{array}{c} C^{1}\\ \vdots \\ C^{M} \end{array}\right)\;{\vec{\lambda }}_{\left(M,1\right)}=\left(\begin{array}{c} \lambda^{1}\\ \vdots \\ \lambda^{M} \end{array}\right)$$

(A2) $$M_{\left(N,N\right)}=\left(\begin{array}{cccccc} 4 & -2 & 0 & \cdots & 0\\ -2 & 4 & -2 & \cdots & 0\\ 0 & -2 & 4 & \cdots & 0\\ \; & \; & \vdots & \; & & \\ 0 & \cdots & -2 & 4 & -2\\ 0 & \cdots & 0 & -2 & 4 \end{array}\right),\;{\vec{I}}_{\left(N,1\right)}=\left(\begin{array}{c} i_{1}\\ \vdots \\ i_{N} \end{array}\right)$$

### Appendix A.2. Solving the Optimization Problem for Lagrange Multipliers

In the general case, we can say that

(A3) $$L=\sum_{n=1}^{N}{\left(i_{n}-i_{n+1}\right)}^{2}+\sum_{m=1}^{M}\lambda_{m}\left(C_{m}-\sum_{n=1}^{N}i_{n}c_{m,n}\right)$$

and for our case, we say that B,G,R are the input digital values for the colors blue, green, and red, respectively. We shall define the following cost function:

$$\begin{array}{c} L=\sum_{n=1}^{N}{\left(i_{n}-i_{n+1}\right)}^{2}+\lambda_{b}\left(B-\sum_{n=1}^{N}i_{n}b_{n}\right)+\lambda_{g}\left(G-\sum_{n=1}^{N}i_{n}g_{n}\right)+\lambda_{r}\left(R-\sum_{n=1}^{N}i_{n}r_{n}\right) \end{array}$$

While $\lambda_{b},\lambda_{g},\lambda_{r}$ are Lagrange Multipliers. The derivative of the cost function with respect to the different parameters is

(A4) $$\frac{\partial L}{\partial \lambda_{b}}=0\Rightarrow B=\sum_{n=1}^{N}i_{n}b_{n}$$

(A5) $$\frac{\partial L}{\partial \lambda_{g}}=0\Rightarrow G=\sum_{n=1}^{N}i_{n}g_{n}$$

(A6) $$\frac{\partial L}{\partial \lambda_{r}}=0\Rightarrow R=\sum_{n=1}^{N}i_{n}r_{n}$$

∀0<k<N:

$$\begin{array}{c} \frac{\partial L}{\partial i_{k}}=2\left(i_{k}-i_{k+1}\right)-2\left(i_{k-1}-i_{k}\right)-\lambda_{b}b_{k}-\lambda_{g}g_{k}-\lambda_{r}r_{k}=0\\ \Rightarrow 4i_{k}-2i_{k+1}-2i_{k-1}=\lambda_{b}b_{k}+\lambda_{g}g_{k}+\lambda_{r}r_{k} \end{array}$$

We would like to write these system of equations as vectors. Hence, we define the following:

(A7) $${\vec{C}}_{\left(3,1\right)}=\left(\begin{array}{c} B\\ G\\ R \end{array}\right)\;{\vec{\lambda }}_{\left(3,1\right)}=\left(\begin{array}{c} \lambda_{b}\\ \lambda_{g}\\ \lambda_{r} \end{array}\right)\;{\vec{I}}_{\left(N,1\right)}=\left(\begin{array}{c} i_{1}\\ i_{2}\\ \vdots \\ i_{N} \end{array}\right)$$

(A8) $$S_{\left(N,3\right)}=\left(\begin{array}{ccc} \vec{b} & \vec{g} & \vec{r} \end{array}\right)\;M_{\left(N,N\right)}=\left(\begin{array}{cccccc} 4 & -2 & 0 & \cdots & 0\\ -2 & 4 & -2 & \cdots & 0\\ 0 & -2 & 4 & \cdots & 0\\ \; & \; & \vdots & \; & & \\ 0 & \cdots & -2 & 4 & -2\\ 0 & \cdots & 0 & -2 & 4 \end{array}\right)$$

(A9) $$B={\vec{b}}^{⊺}\cdot \vec{I}\;G={\vec{g}}^{⊺}\cdot \vec{I}\;R={\vec{r}}^{⊺}\cdot \vec{I}\;M\cdot \vec{I}=S\cdot \vec{\lambda }\;$$

Notice that M is a full-rank matrix (adding every row half from the row above leads to an upper triangular matrix), so we can say

$$\begin{array}{cc} \vec{I}=M^{-1}S\cdot \vec{\lambda }\Rightarrow \; & B={\vec{b}}^{⊺}\cdot M^{-1}S\cdot \vec{\lambda }\\ \; & G={\vec{g}}^{⊺}\cdot M^{-1}S\cdot \vec{\lambda }\\ \; & R={\vec{r}}^{⊺}\cdot M^{-1}S\cdot \vec{\lambda } \end{array}$$

and we can write this as

(A10) $$\vec{C}=S^{⊺}M^{-1}S\cdot \vec{\lambda }$$

By requiring that *S* needs to be a full-rank matrix and inverting the relation to find Lagrange multipliers, we find

(A11) $$\vec{\lambda }={\left(S^{⊺}M^{-1}S\right)}^{-1}\cdot \vec{C}$$

and the final result is

(A12) $$\vec{I}=M^{-1}S{\left(S^{⊺}M^{-1}S\right)}^{-1}\cdot \vec{C}$$

This compact result provides us a way (given flicker vectors of $\vec{b},\vec{g},\vec{r}$) to extract the signal values into each frame $\vec{I}$ using the total input values of blue, green, and red intensity. Note that we required the *S* matrix, which represents the flicker pattern, to be a full-rank matrix. This requirement limits the number of possible matrices, assuming $S_{ij}$ can only be 0 or 1. In general, it is not obvious what *S* matrix should be under these constraints because it very much depends on the sampled function behavior manner. We will present an analysis regarding choosing the flicker pattern later.

### Appendix A.3. Expanding to Spatial Regularization

To enhance the spatial correlation between adjacent pixels, the cost function can be modified to add some spatial regularization. We assume that only the first-level neighbors are relevant, and we define the domain P to be a five-pixel domain (see Figure A1).

$$\begin{array}{c} L=\sum_{n=1}^{N-1}\sum_{x,y\in P}w_{x,y}^{t}{\left(i_{x,y,n}-i_{x,y,n+1}\right)}^{2}+w_{x,y}^{s}{\left(i_{x,y,n}-i_{x,y+1,n}\right)}^{2}+\\ w_{x,y}^{s}{\left(i_{x,y,n}-i_{x+1,y,n}\right)}^{2}+\sum_{m=1}^{M}\sum_{x,y\in Patch}\lambda_{m,x,y}\left(C_{m,x,y}-\sum_{n=1}^{N}i_{x,y,n}c_{x,y,m,n}\right) \end{array}$$

where $w_{x,y}^{t},w_{x,y}^{s}$ are the weight factors for the temporal condition and the spatial condition, respectively. For simplicity, we assume a constant weight for $w_{x,y}^{s}=w^{s},w_{x,y}^{t}=w^{t}$.

This system has the same solution form when mapping the different vectors to make column stack vectors (for each of the pixels); for example,

(A13) $${\vec{C}}_{\left(3*5,1\right)}=\left(\begin{array}{c} B_{1}\\ G_{1}\\ R_{1}\\ \vdots \\ B_{5}\\ G_{5}\\ R_{5} \end{array}\right)\;{\vec{\lambda }}_{\left(3*5,1\right)}=\left(\begin{array}{c} \lambda_{b,1}\\ \lambda_{g,1}\\ \lambda_{r,1}\\ \vdots \\ \lambda_{b,5}\\ \lambda_{g,5}\\ \lambda_{r,5} \end{array}\right)$$

The S matrix (5N × 15) is changed into a block diagonal matrix, while each block corresponds to different pixels in the domain P. M matrix (5N × 15) has changed to be

(A14) $$M_{i,j}=\left\{\begin{array}{cc} 2w^{s}+2w^{t} & \mathrm{if}\;i=j\\ -2w^{t} & \mathrm{if}\;|i-j|=1\\ -2w^{s} & \mathrm{if}\;|i-j|mod5=0\\ 0 & \mathrm{else}\; \end{array}\right.$$

### Appendix A.4. Signals Similarity Metrics

To evaluate the method’s accuracy, we shall compare our result with the actual signal. Since we deal with the function for finite energy (in $L^{2}$), the inner product is defined as

(A15) $$<f_{1}\left(t\right),f_{2}\left(t\right)>\equiv \int_{-\infty }^{\infty }f_{1}\left(t\right)f_{2}\left(t\right)dt$$

We introduce two error metrics that we found to be the most reasonable to examine:

Euclidean distance (L2):

(A16) $$L_{2}\left(f_{1}\left(t\right),f_{2}\left(t\right)\right)\equiv \sqrt{\left|\right|f_{1}\left(t\right)-f_{2}\left(t\right){\left|\right|}_{2}^{2}}$$

Cosine similarity:

(A17) $$Error\left(f_{1}\left(t\right),f_{2}\left(t\right)\right)\equiv \cos^{-1}\left(\frac{<f_{1}\left(t\right),f_{2}\left(t\right)>}{\sqrt{\left|\right|f_{1}{\left(t\right)||}^{2}\left|\right|f_{2}\left(t\right){\left|\right|}^{2}}}\right)$$

The first metric was chosen because it is a prevalent and intuitive way to compare signals. The second metric was chosen to avoid bias due to the signals’ absolute magnitude and to focus only on their shape relations; it was used in the experimental part.

### Appendix A.5. Flicker Order Choices

N = 4:

$$\begin{array}{cc} 0:\; & \vec{b}=\left(1,0,0,1\right),\vec{g}=\left(0,1,0,0\right),\vec{r}=\left(0,0,1,0\right)\\ 1:\; & \vec{b}=\left(1,0,0,1\right),\vec{g}=\left(1,0,1,0\right),\vec{r}=\left(0,1,0,1\right)\\ 2:\; & \vec{b}=\left(1,0,0,0\right),\vec{g}=\left(0,1,1,0\right),\vec{r}=\left(0,0,0,1\right)\\ 3:\; & \vec{b}=\left(1,1,0,0\right),\vec{g}=\left(0,1,1,0\right),\vec{r}=\left(0,0,1,1\right)\\ 4:\; & \vec{b}=\left(0,0,1,0\right),\vec{g}=\left(0,1,0,0\right),\vec{r}=\left(1,1,1,1\right) \end{array}$$

N = 5:

$$\begin{array}{cc} 0:\; & \vec{b}=\left(1,0,0,0,1\right),\vec{g}=\left(0,1,1,0,0\right),\vec{r}=\left(0,0,1,1,0\right)\\ 1:\; & \vec{b}=\left(1,0,0,1,0\right),\vec{g}=\left(1,0,1,0,1\right),\vec{r}=\left(0,1,0,0,1\right)\\ 2:\; & \vec{b}=\left(1,0,0,1,0\right),\vec{g}=\left(0,0,1,0,0\right),\vec{r}=\left(0,1,0,0,1\right)\\ 3:\; & \vec{b}=\left(0,1,0,0,0\right),\vec{g}=\left(1,0,1,0,1\right),\vec{r}=\left(0,0,0,1,0\right)\\ 4:\; & \vec{b}=\left(1,1,0,0,0\right),\vec{g}=\left(0,1,1,1,0\right),\vec{r}=\left(0,0,0,1,1\right) \end{array}$$

N = 6:

$$\begin{array}{cc} 0:\; & \vec{b}=\left(1,0,0,0,1,0\right),\vec{g}=\left(0,1,0,0,0,1\right),\vec{r}=\left(0,0,1,1,0,0\right)\\ 1:\; & \vec{b}=\left(1,1,0,0,0,0\right),\vec{g}=\left(0,0,1,1,0,0\right),\vec{r}=\left(0,0,0,0,1,1\right)\\ 2:\; & \vec{b}=\left(1,0,0,1,0,0\right),\vec{g}=\left(0,1,1,1,1,0\right),\vec{r}=\left(0,0,1,0,0,1\right)\\ 3:\; & \vec{b}=\left(1,0,1,0,1,0\right),\vec{g}=\left(0,1,0,1,0,1\right),\vec{r}=\left(1,1,1,1,1,1\right)\\ 4:\; & \vec{b}=\left(0,1,0,0,0,0\right),\vec{g}=\left(1,0,1,1,0,1\right),\vec{r}=\left(0,0,0,0,1,0\right) \end{array}$$

### Appendix A.6. SNR Analysis

The Signal: We split the exposure time to N disjoint time steps according to the derivation. At the same time, for each of these steps, we assumed that the captured light was generated by the light source and then reflected from the object (up to a color reflective factor), in addition to the environment’s existing background illumination. In that case, we can not assume that the captured signal was taken only at the time steps when the flicker was applied. Therefore, a more precise derivation should be presented (we denote *b* = blue, *g* = green, and *r* = red, and *t* is the exposure time of the camera):

$$\begin{array}{cc} S_{TSR}= & \sum_{n=1}^{N}\frac{t}{N}\left[\gamma_{b}\left(i_{n}^{b\;env}+i_{n}^{b\;flicker}\right)+\gamma_{g}\left(i_{n}^{g\;env}+i_{n}^{g\;flicker}\right)+\gamma_{r}\left(i_{n}^{r\;env}+i_{n}^{r\;flicker}\right)\right] \end{array}$$

This equation says that, for each frame, the signal comprises a sum of the intensity from the environment and the flicker over *N* time steps. We assume that the flickering intensity is equal for different colors, and we denote it as $i^{flicker}$. We assume that the environment light is a constant white light with approximately equal intensity for the entire spectral range, denoting it as $i^{env}$. In addition, we denote the ratio $\alpha \equiv \frac{i^{flicker}}{i^{env}}$ so we can say that

$$\begin{array}{c} S_{TSR}\approx \left(\alpha^{-1}+1\right)i^{flicker}\sum_{n=1}^{N}\frac{t}{N}\left(\gamma_{b}b_{n}+\gamma_{g}g_{n}+\gamma_{r}r_{n}\right)\geq \left(\frac{1}{\alpha }+1\right)i^{flicker}t\min\left\{\gamma_{b},\gamma_{g},\gamma_{r}\right\} \end{array}$$

We used the fact that we do not allow a time step without a flicker at all. The signal ratio between the signal with and without the flicker ($S_{T}$ represents the SNR without the flicker):

$$\begin{array}{c} \frac{S_{TSR}}{S_{T}}=\frac{\left(\alpha^{-1}+1\right)i^{flicker}\sum_{n=1}^{N}\frac{t}{N}\left(\gamma_{b}b_{n}+\gamma_{g}g_{n}+\gamma_{r}r_{n}\right)}{ti^{env}}\geq \left(1+\alpha \right)\min\left\{\gamma_{b},\gamma_{g},\gamma_{r}\right\} \end{array}$$

The Noise: Say that we deal with a temporal signal and separate its exposure time into several temporal steps. Then, we adopt the noise model, as presented in [28]. Note that the sensor still works on the same original exposure time, but we assume that the dominant noise factor for the source illumination is the shot noise:

(A18) $$Noise_{TSR}=\sqrt{S_{TSR}+Dt+N_{r}^{2}}\approx \sqrt{S_{TSR}}$$

while *t* is the camera’s total exposure time, *D* represents the dark noise coefficient factor, and $N_{r}$ the read noise. Then, we can say that the noise ratio between the signals is

(A19) $$\frac{Noise_{TSR}}{Noise_{T}}=\sqrt{\frac{Signal_{TSR}}{Signal_{T}t+Dt+N_{r}^{2}}}\leq \sqrt{\frac{Signal_{TSR}}{S_{T}}}$$

The SNR ratio: According to the definition:

$$\begin{array}{cc} \frac{SNR_{TSR}}{SNR_{T}}= & \frac{\frac{S_{TSR}}{Noise_{TSR}}}{\frac{S_{T}}{Noise_{T}}}\geq {\left(\frac{S_{TSR}}{S_{T}}\right)}^{3/2}\geq {\left[\left(1+\alpha \right)\min\left\{\gamma_{b},\gamma_{g},\gamma_{r}\right\}\right]}^{3/2} \end{array}$$

This means that the SNR improves significantly for increasing the α factor. For an approximately white object and when α>>1 (strong flicker), we find that the improvement in the SNR is $\propto \alpha^{3/2}$.

Dealing with environmental illumination: When the environmental illumination is not negligible (α∼1), the previous assumption about the detected light does not hold anymore. Then, we can estimate the error in our method, saying that each channel has an additional detected light from the environment:

(A20) $$\vec{C}\Rightarrow \vec{C}+\Delta \vec{C}=\vec{C}+\frac{1}{\alpha }\vec{C}$$

which leads to a change in ΔI:

(A21) $$\vec{I}\Rightarrow \vec{I}+\Delta \vec{I}$$

(A22) $$\Delta \vec{I}=\frac{1}{\alpha }M^{-1}S{\left(S^{⊺}M^{-1}S\right)}^{-1}\cdot \vec{C}=\alpha^{-1}\vec{I}$$

Therefore, the error grows as $\alpha^{-1}$. It is worth mentioning that the contribution of the illumination of the environment can be estimated before and then subtracted from the channel vectors.

### Appendix A.7. Regularization Analysis

A comparison for different frame reconstructions given different regularization (spatial vs. temporal) is presented in Figure A4:
